# Mixed Nuts as Healthy Snacks: Effect on Tryptophan Metabolism and Cardiovascular Risk Factors

**DOI:** 10.3390/nu15030569

**Published:** 2023-01-21

**Authors:** Jieping Yang, Rupo Lee, Zachary Schulz, Albert Hsu, Jonathan Pai, Scarlet Yang, Susanne M. Henning, Jianjun Huang, Jonathan P. Jacobs, David Heber, Zhaoping Li

**Affiliations:** 1 Center for Human Nutrition, David Geffen School of Medicine at UCLA, Los Angeles, CA 90095, USA; 2 The Vatche and Tamar Manoukian Division of Digestive Diseases, Department of Medicine, David Geffen School of Medicine at UCLA, Los Angeles, CA 90095, USA; 3 Division of Gastroenterology, Hepatology and Parenteral Nutrition, Veterans Affairs Greater Los Angeles Healthcare System, Los Angeles, CA 90073, USA; 4 Department of Medicine, VA Greater Los Angeles Health Care System, Los Angeles, CA 90073, USA

**Keywords:** tree nuts, cardiovascular risk, tryptophan metabolism, gut microbiome, weight loss

## Abstract

We recently demonstrated that the consumption of mixed tree nuts (MTNs) during caloric restriction decreased cardiovascular risk factors and increased satiety. Tryptophan (Trp) metabolism has been indicated as a factor in cardiovascular disease. Here, we investigated the effect of MTNs on Trp metabolism and the link to cardiovascular risk markers. Plasma and stool were collected from 95 overweight individuals who consumed either MTNs (or pretzels) daily as part of a hypocaloric weight loss diet for 12 weeks followed by an isocaloric weight maintenance program for an additional 12 weeks. Plasma and fecal samples were evaluated for Trp metabolites by LC–MS and for gut microbiota by 16S rRNA sequencing. Trp–kynurenine metabolism was reduced only in the MTNs group during weight loss (baseline vs. week 12). Changes in Trp–serotonin (week 24) and Trp–indole (week 12) metabolism from baseline were increased in the MTNs group compared to the pretzel group. Intergroup analysis between MTN and pretzel groups does not identify significant microbial changes as indicated by alpha diversity and beta diversity. Changes in the relative abundance of genus *Paludicola* during intervention are statistically different between the MTNs and pretzel group with *p* < 0.001 (*q* = 0.07). Our findings suggest that consumption of MTNs affects Trp host and microbial metabolism in overweight and obese subjects.

## 1. Introduction

Tree nuts are an excellent source of protein, unsaturated fatty acids, fiber, minerals, vitamins, and phytochemicals [1]. Tree nut consumption has been associated with many health benefits including decrease in markers of cardiovascular disease, metabolic syndrome, and body weight [2]. Due to their fiber and polyphenol content, tree nuts have been suggested to have prebiotic activity and affect human health partially through modulating gut microbiome composition, such as increasing alpha microbial diversity and reducing pathogenic bacteria [3,4,5,6]. Recent studies showed that the anti-oxidant capacity of phenolic compounds are positively associated with their microbial modulation potential [7]. The alteration in gut microbiota leads to changes in microbial metabolites in the intestine and systemic blood. For example, Holscher et al. demonstrated an effect of walnut nut consumption on microbial bile acid metabolism [8].

Tryptophan (Trp) is an essential amino acid, and tree nuts are a high Trp dietary source. Trp metabolism is involved in many aspects of host metabolism and physiology [9,10]. Trp metabolism in hosts occurs via the kynurenine (KYN) pathway or the serotonin pathway to produce bioactive metabolites. In the intestine, Trp is metabolized by gut microbes into indole and indole derivatives [11]. The majority of Trp is metabolized through the KYN pathway by host cells, which generates many bioactive metabolites that are important for immune regulation [3]. Alterations in the KYN pathway have been previously reported in diabetes and insulin resistance, cardiovascular diseases (CVD), and an increase in the KYN/Trp ratio is often associated with immune activation [12,13,14]. Trp is also metabolized into neuroactive metabolites, such as serotonin, by host cells, and indole derivatives by gut microbiota [15]. About 90% of serotonin in the body is formed in gut cells and released into the blood stream. This process is regulated by gut bacteria [16]. The immunomodulatory role of circulating serotonin is well-established [17]. Microbial indole metabolites of Trp, such as indole-3-propionic acid (IPA), have been associated with decreased risk of cardiovascular disease and type 2 diabetes, and anti-inflammatory and neuroprotection effects [9,18,19,20]. A previous study showed that the consumption of a Mediterranean diet including nuts compared to a fast-food Western diet was associated with an increase in serum Trp metabolites, IPA, and indole-3-lactic acid [21]. 

In our previous randomized, controlled, two-arm study, we demonstrated that the consumption of 1.5 oz of mixed tree nuts (MTNs) as part of hypocaloric diet (500 kcal per day less than resting metabolic rate) for 12 weeks resulted in weight loss, increased satiety, decreased diastolic blood pressure, and decreased heart rate, but did not change lipid markers [22]. It is possible that changes in the gut microbial composition and microbial metabolites in systemic circulation might link nut consumption to improved cardiovascular markers. Therefore, it is the aim of the present study to utilize fecal and blood samples from this previous study and investigate whether snacks of MTNs as part of a hypocaloric diet modify the gut microbiome, leading to an increase in levels of cardio-protective Trp microbial metabolites.

## 2. Materials and Methods

### 2.1. Study Design

We recently evaluated whether incorporating MTNs (*n* = 56) in a calorie-restricted weight loss and weight maintenance program lead to weight loss and reduced inflammation compared to pretzel control (*n* = 39) in a randomized, controlled, parallel, two-arm study [22]. The clinical protocol was approved by the Internal Review Board of the University of California, Los Angeles. All subjects provided written informed consent before the study began. The study was registered in ClinicalTrials.gov under the following identifier: NCT03159689. This study was performed at the UCLA Center for Human Nutrition CA in accordance with the guidelines of the Human Subjects Protection Committee of the University of California, Los Angeles. Participants were randomized to either 1.5 oz mixed tree nuts (MTN) or pretzels with equal caloric content daily during weight loss (−500 kcal hypocaloric diet) for 12 weeks and isocaloric diet for another 12 weeks of weight maintenance. Participants’ characteristics at baseline was shown in Supplementary Table S1. Fecal and blood samples were collected at baseline, 12, and 24 weeks from 56 participants in the MTN group and 39 participants in the pretzel group [22]. Stool samples from 56 participants in the MTN group and 39 participants in the pretzel group were used for microbiota analysis. Plasma and stool samples from 56 participants in the MTN group and 38 participants in the pretzel group used for tryptophan (Trp) metabolite analysis. Plasma samples from 1 subject from the pretzel group were not enough to conduct the analysis.

### 2.2. Measurement of Plasma and Stool Trp and Its Major Metabolites

Plasma samples were prepared as previously described [23]. Stool samples were suspended in methanol water (4:1) with 1% ascorbic acid at the concentration of 10 µL/mg, vortexed and sonicated for 10 min at 4 °C. Samples were then centrifuged at 12,000× *g* for 10 min at 4 °C. Trp and its metabolites (indole-acetic acid (IAA), kynurenine (KYN), kynurenic acid (KYNA), indole-3-propionatic acid (IPA), indole, serotonin, 3-methylindole, tryptamine, and indole sulfate (IS)) were analyzed using liquid chromatography coupled to electrospray ionization triple quadrupole mass spectrometry (LC–ESI–MS/MS) and HPLC fluorescence as previously described [23].

### 2.3. Fecal 16S rRNA Gene Sequencing and Data Analysis

DNA extraction and sequencing of the 16S rRNA gene were performed as previously described by UCLA Microbiome Core [24]. Fecal bacterial DNA was extracted using the ZymoBIOMICS DNA Miniprep Kit. The V4 region of the 16S gene was amplified and barcoded using 515f/806r primers, then 250 × 2 bp sequencing was performed on an Illumina MiSeq. Amplicon sequence variants (ASVs) were identified using DADA2 and annotated against the SILVA v138 database. Alpha diversity metrics (Chao1 and Shannon index) were calculated and tested for significance using linear mixed effects models (alpha diversity ~ age + gender + race + intervention + time + intervention * time) with subjects as random effects. All modelling was performed using the *lmerTest* R package [25]. Beta diversity was calculated using Bray–Curtis dissimilarity and analyzed by repeated measures permutational multivariate analysis of variance (PERMANOVA) using omnibus and Vegan in R, and was visualized via principal coordinate analysis (PCoA) ordination as previously described [26,27]. We performed per-feature testing in multivariate association with linear models MaAsLin2 in R package using linear mixed effects models to examine the potential association between genera with intervention, time and interaction between intervention and time or between genera and Trp metabolites [28]. These models accounted for within-individual correlation from the study’s repeated sampling design. All detected associations were adjusted for subjects as random effect and other fixed effects metadata including age, gender, race (reference level: white), intervention (reference level: pretzel), time (reference level: W0), and intervention and time interaction. The current analysis was performed after filtering at minimum abundance level of 0.0001 and minimum prevalence of 0.2. The relative abundance of genera was log transformed. Only significant associations with *q* ≤ 0.25 after a false discovery rate (FDR) correction were included.

### 2.4. Statistical Analysis

To detect difference in changes in Trp metabolites between groups (NUTS vs. PRETZEL) over time, we built linear mixed models that included fixed effects of intervention, time, intervention–time, age, sex, race, and subject as random effects. Between-group comparison had intervention, time, and intervention–time interactions to estimate the intervention effects at different time points. Within-group comparison had the time variable to estimate the change over time. Values of *p* < 0.05 were considered statistically significant. All statistical analysis was performed using the *lmerTest* R package [25].

## 3. Results

### 3.1. The Association between Trp Metabolites and Cardiovascular Risk Factors

**Figure 1:**
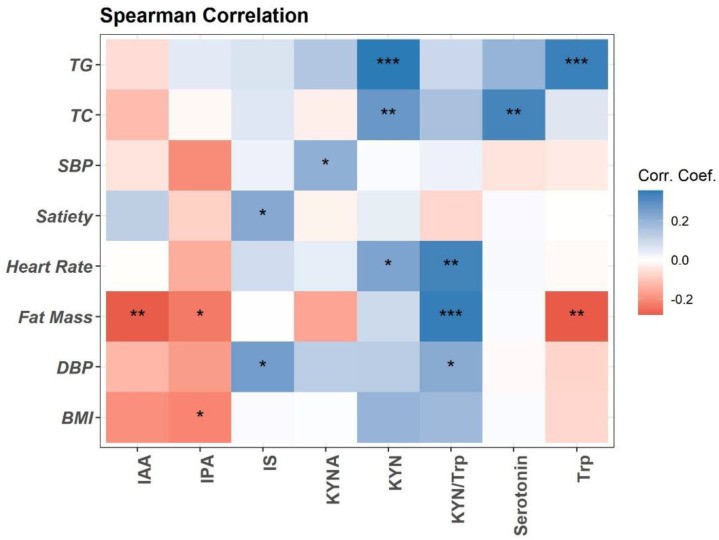
Spearman correlation of plasma Trp metabolites with clinical measurements at baseline visit (*n* = 94). Heatmap depicting the Spearman correlation patterns (* *p* < 0.05, ** *p* < 0.01, *** *p* < 0.001. Body mass index (BMI), systolic and diastolic blood pressure (SBP, DBP), total cholesterol (TC), total triglycerides (TG), indole-acetic acid (IAA), kynurenine (KYN), kynurenic acid (KYNA), indole-3-propionatic acid (IPA), and indole sulfate (IS).

Trp metabolism is altered in obesity and associated with obesity-related inflammation [11,29]. Here, we performed the Spearman association analysis between baseline plasma concentrations of Trp metabolites and clinical variables (Figure 1). Briefly, plasma Trp, IPA, and IAA are negatively associated with fat mass and BMI. Trp is positively associated with blood total triglycerides (TG). Trp metabolism towards the KYN pathway (KYN, KYA, and KYN/Trp ratio) is positively associated with fat mass, heart rate, blood pressure (DBP), and lipids (TG and total cholesterol (TC)) (Figure 1). Plasma Trp microbial metabolite IS is positively associated with DBP and satiety (Figure 1). Positive association is detected between plasma serotonin and TC (Figure 1).

### 3.2. Effects of MTNs Consumption on Trp Metabolism

**Figure 2:**
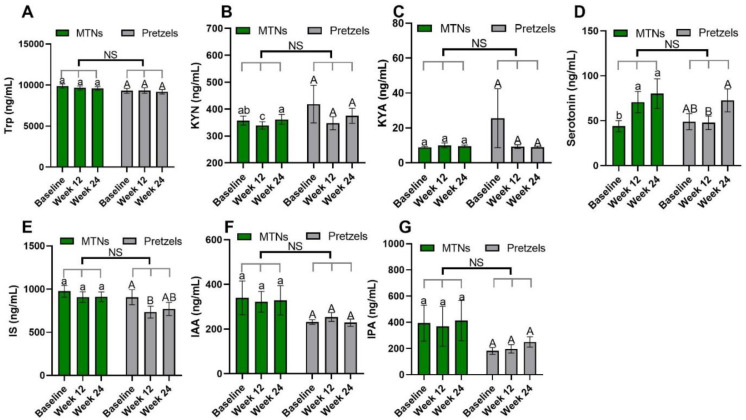
Levels of plasma Trp (**A**) and Trp metabolites, (**B**) KYN, (**C**) KYNA, (**D**) serotonin, (**E**) IS, (**F**) IAA, and (**G**) IPA at baseline, week 12, and week 24 in pretzel group (*n* = 38) and MTNs group (*n* = 56). The results are presented as mean values with SEM. For within-group analysis (lower case for MTNs; upper case for pretzels), means in a column without a common letter differ; *p* < 0.05. Changes from baseline were analyzed and no between-groups significant difference (NS) was detected.

Trp–KYN metabolism is associated with the pathogenesis of CVD [13,30]. Compared to baseline, plasma KYN concentrations are significantly reduced in the MTNs group at the end of calorie-restricted weight loss (week 12) and return to baseline concentrations after 12 weeks of isocaloric weight maintenance (week 24), while remaining at similar levels during weight loss and weight maintenance in the pretzel group (Figure 2B).

**Figure 3:**
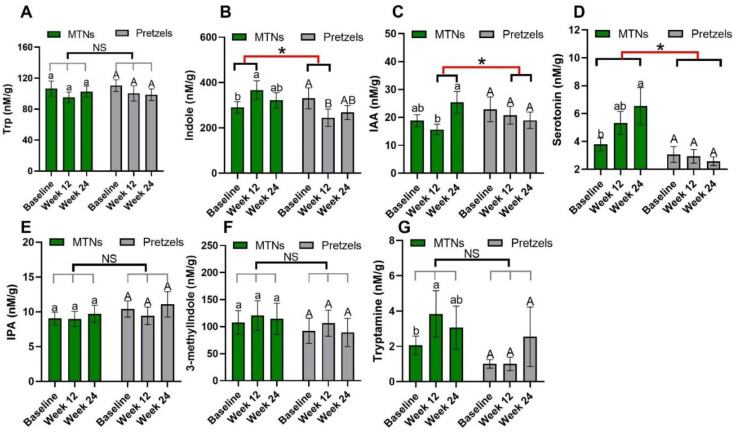
Levels of fecal Trp (**A**) and Trp metabolites, (**B**) KYN, (**C**) KYNA, (**D**) serotonin, (**E**) IS, (**F**) IAA, and (**G**) IPA at baseline, week 12, and week 24 in pretzel group (*n* = 39) and MTNs group (*n* = 56). The results are presented as mean values with SEM. For within-group analysis (lower case for MTNs; upper case for pretzels), means in a column without a common letter differ, *p* < 0.05. Changes from baseline were analyzed and between-groups difference is significant * when *p* < 0.05. NS, no significant difference between-groups.

Trp–serotonin metabolism was evaluated by measuring plasma and fecal serotonin levels during intervention. Plasma serotonin concentrations increase significantly with the consumption of MTNs in both weight loss (week 12) and maintenance (week 24) periods, but only increase at the end of weight maintenance (week 24) compared to week 12 in the pretzel group (Figure 2D). Fecal serotonin levels increase gradually with MTNs consumption (Figure 3D). Changes in serotonin at the end of isocaloric weight maintenance (week 24) from baseline are significantly different between the MTNs and pretzel groups (Figure 3D).

Trp–indole metabolism was evaluated by measuring plasma IS, IAA, IPA and fecal indole, IAA, IPA, 3-methylindole, and tryptamine. In both the MTNs and pretzel groups, plasma Trp, IAA, and IPA, as well as fecal Trp, IPA, and 3-methylindole concentrations, remain unchanged during the 24 weeks intervention (Figure 2A,F,G, and Figure 3A,E,F). Plasma IS concentration is found to be significantly reduced at the end of weight loss (week 12) in the pretzel group, but not in the MTNs group (Figure 2E). Fecal indole and tryptamine levels are significantly increased in the MTNs group at the end of calorie-restricted weight loss (week 12) and return to baseline levels after 12 weeks of isocaloric weight maintenance (week 24) (Figure 3B,G). Changes in indole at the end of weight loss from baseline are significantly different between MTNs and Pretzels groups (Figure 3B). Fecal IAA levels are increased in the MTNs group at the end of weight maintenance (week 24) and changes in IAA at the end of isocaloric weight maintenance (week 24) from the end of weight loss (week 12) are significantly different between the MTNs and pretzel groups (Figure 3C).

### 3.3. Effects of MTNs and Pretzel Consumption on Fecal Microbiome

**Figure 4:**
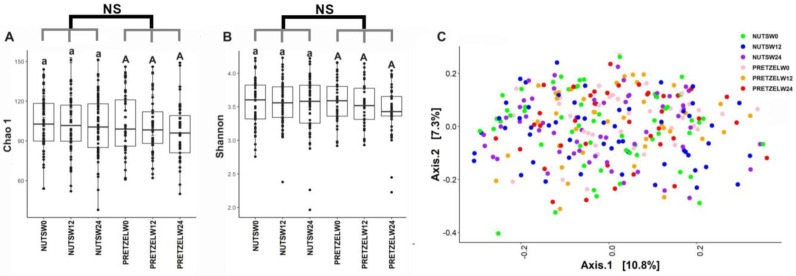
Gut microbiome diversity analysis. Alpha diversity indexes (**A**) Chao 1 and (**B**) Shannon and (**C**) principal coordinate analysis plot of *β*-diversity measure Bray–Curtis dissimilarity plot of all fecal samples from pretzel group (*n* = 39) and MTNs group (*n* = 56) collected at baseline (PRETZELW0, NUTSW0), week 12 (PRETZELW12, NUTSW12), and week 24 (PRETZELW24, NUTSW24). For within-group analysis of alpha diversity (lower case for MTNs; upper case for pretzels), means in a column without a common letter differ, *p* < 0.05). Changes from baseline were analyzed and no between-groups significant difference (NS) was detected.

Fecal microbiome was evaluated in all participants at baseline, and the end of 12 weeks of calorie-restricted weight loss and 12 weeks of weight maintenance. There is no significant difference between the MTNs and pretzel groups during 24 weeks in alpha diversity, as indicated by Chao1 and Shannon indices (Figure 4A,B). Beta diversity analysis, as indicated by Bray–Curtis distance, does not show a distinct separation between baseline, week 12, and week 24 in either the MTNs or pretzel group (Figure 4C).

**Figure 5:**
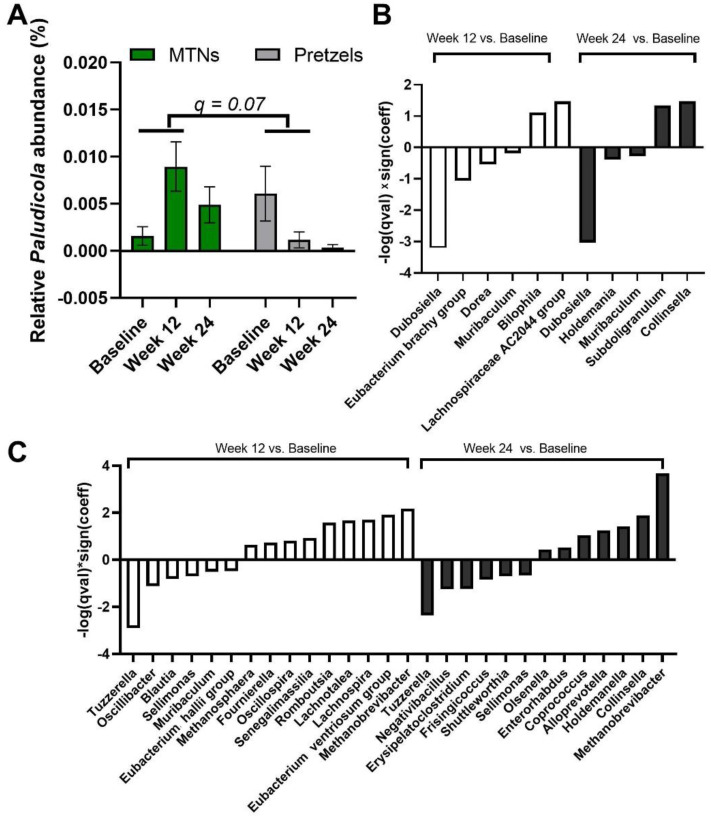
MaAsLin2 significant results at genus level. (**A**) Relative abundance of genus *Paludicola* at baseline, week 12, and 24. Significant association is observed between (interaction: week 12 and intervention) and relative abundance of *Paludicola* at the genus level. *q* value < 0.25 is considered significant. Significant association (*q* < 0.25) between time and gut microbiome composition at genus level in (**B**) MTNs and (**C**) pretzel groups. Sign(coeff) refers to the absolute value of the coefficients in the MaAsLin2 model. −log(*q* value) × Sign(coeff) is calculated to emphasize associations with both large effects and high statistical significance.

We also examined the multivariable association between bacterial genera and intervention–time. At genus level, *Paludicola* relative abundance is increased in fecal samples collected at week 12 from subjects in the MTNs group compared to samples collected at week 12 from subjects in the pretzel group, with *p* < 0.001 (*q* = 0.07) (Figure 5A). In MTNs, the relative abundance of *Dubosiella*, *Eubacterium brachy group*, *Dorea*, and *Muribaculum* is reduced, while relative abundance of *Bilophila* and *Lachnospiraceae AC2044 group* is increased at the end of weight loss (week 12) compared to baseline (Figure 5B); relative abundance of *Dubosiella*, *Holdemania*, and *Muribaculum* is reduced, while relative abundance of *Subdoligranulum* and *Collinsella* is increased at the end of weight maintenance (week 24) compared to baseline (Figure 5B). In the pretzel group, the relative abundance of *Tuzzerella*, *Oscillibacter*, *Blautia*, *Sellimonas*, *Muribaculum*, and *Eubacterium hallii group* is reduced, while relative abundance of *Methanosphaera*, *Fournierella*, *Oscillospira*, *Senegalimassilia*, *Romboutsia*, *Lachnotalea*, *Lachnospira*, *Eubacterium ventriosum group*, and *Methanobrevibacter* is increased at the end of weight loss (week 12) compared to baseline (Figure 5C); relative abundance of *Tuzzerella, Negativibacillus*, *Erysipelatoclostridium*, *Frisingicoccus*, *Shuttleworthia*, and *Sellimonas* is reduced, while relative abundance of *Olsenella*, *Enterorhabdus*, *Coprococcus*, *Alloprevotella*, *Holdemanella*, *Collinsella*, and *Methanobrevibacter* is increased at the end of weight maintenance (week 24) compared to baseline (Figure 5C).

### 3.4. Association between Trp Metabolism and Gut Microbiome

**Figure 6:**
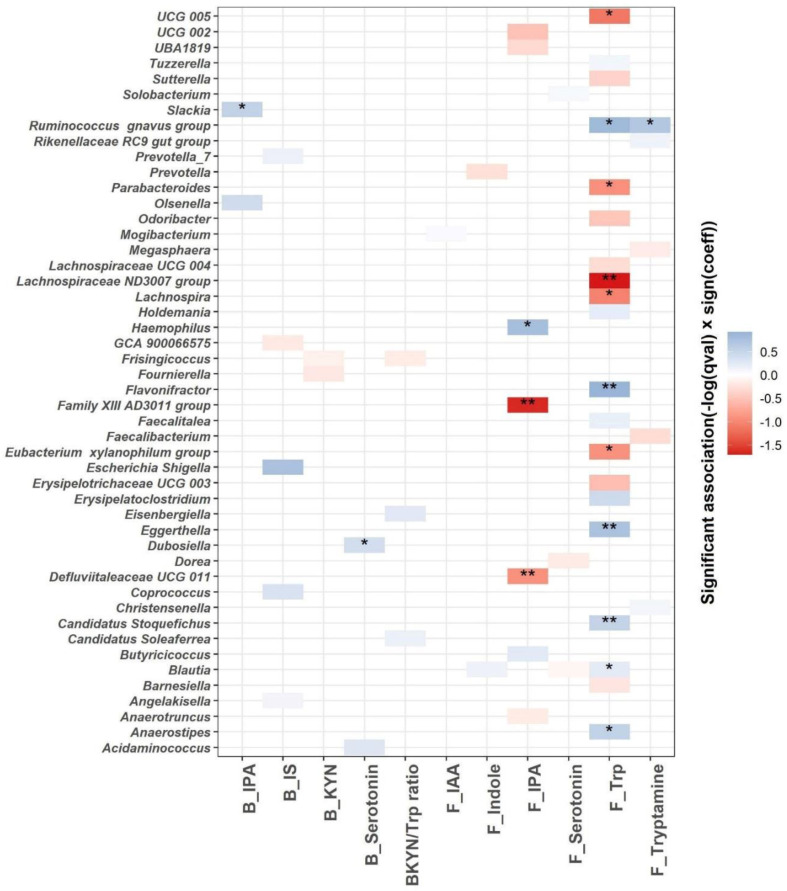
MaAsLin2 significant associations (−log(*q* value) × sign(coeff) between blood, fecal Trp metabolites (blood IPA (B_IPA); blood IS (B_IS); blood KYN (B_KYN); blood serotonin (B_serotonin); blood KYN/Trp ratio (BKYN/Trp ratio); fecal IAA (F_IAA); fecal indole (F_Indole); fecal serotonin (F_Serotonin); fecal Trp (F_Trp) and fecal tryptamine (F_Tryptamine))and gut microbial composition at the genus level across all samples after adjusting for age, gender, race, intervention, time, and intervention–time interaction. Based on normalized obtained significant results, the color scale bar shows a positive relationship (blue) and a negative one (red) between taxa and Trp metabolites. * *q* value < 0.05, ** *q* value < 0.01.

We used linear mixed models to identify microbial genera associated with the Trp metabolism as indicated by blood and fecal Trp metabolites (Figure 6). A total of 48 genera are significantly associated with the blood or fecal Trp metabolites (*q*  ≤  0.25; Figure 6 and Supplementary Table S2). The most significant associations are identified between fecal Trp and microbial genera. A total of 11 out of 20 differentially abundant microbial genera are associated significantly with fecal Trp, with *q* value < 0.05. Among the 11 associations with *q* value < 0.05, fecal Trp is negatively associated with *Lachnospiraceae ND3007 group*, *UCG 005*, *Lachnospira*, *Parabacteroides*, and *Eubacterium xylanophilum group*, and positively associated with *Blautia*, *Candidatus Stoquefichus*, *Anaerostipes*, *Eggerthella*, *Flavonifractor*, and *Ruminococcus gnavus group*. Fecal tryptamine is positively associated with *Ruminococcus gnavus group* (*q* < 0.05), which is in agreement with previous efforts identifying *Ruminococcus gnavus group* as tryptamine producer [31]. A total of three out of seven differentially abundant microbial genera are significantly associated with fecal IPA with *q* value < 0.05. Fecal IPA is negatively associated with *Family XIII AD3011 group* and *Defluviitaleaceae UCG 011*, and positively associated with *Haemophilus* (*q* < 0.05). Blood IPA and serotonin are positively associated with *Slackia* and *Dubosiella*, respectively (*q* < 0.05).

## 4. Discussion

We recently reported that the incorporation of MTNs into a hypocaloric diet shows increased satiety and reduced heart rate, but similar weight loss and decreased DBP when compared to pretzel control [22]. MTNs are characterized as high Trp foods. Trp metabolism is considered a potential pharmacological target, as bioactive molecules produced from Trp metabolism regulate many important biological processes, including gastrointestinal functions, immunity, metabolism, and the nervous system [11]. Dysregulation of Trp metabolism has been reported in metabolic diseases such as obesity and CVD [29,32]. Our finding of negative association between Trp, IPA, and IAA, and positive association between KYN with fat mass and BMI, is consistent with previous publications showing reduced Trp, IPA, and IAA levels but increased KYN levels in obese subjects compared to healthy subjects [20,29]. In the present study, we discover some new associations between Trp metabolites (KYN, KYN/Trp, and KYA) and blood pressure, heart rate, and satiety in overweight/obese subjects, suggesting a broader impact of Trp metabolism in host health, including cardiovascular health. 

During a 24 week MTN intervention, specific changes in the Trp metabolism were observed. Changes in fecal indole levels at the end of weight loss (week 12) compared to baseline are significantly different between the MTNs and pretzel groups. Levels of fecal indole increase in the MTNs group but decrease in the pretzel group, suggesting Trp indole metabolism is reduced during weight loss but reversed by introducing MTNs as a dietary Trp source. About 4–6% of unabsorbed Trp is metabolized by the microbes into indole and indolic compounds. Indole production has an important impact on host health, by promoting intestinal homeostasis and regulating intestinal immune response, as well as providing antioxidants and neuroprotective metabolites [33]. In addition, changes in fecal serotonin at the end of weight maintenance from baseline differs significantly between the MTNs and pretzel groups. Fecal serotonin and tryptamine increase in the MTN group, but remain unchanged in the pretzel group, suggesting MTNs consumption increases dietary Trp intake and leads to increased serotonin and tryptamine production. However, levels of blood serotonin are not only increased with MTN consumption, but also significantly increased at the end of weight maintenance in the pretzel group. Whether the increase in blood serotonin at the end of weight maintenance is one of the physiological outcomes of body recovery responses to weight loss, and whether the higher blood serotonin contributes to the observed increased satiety during weight loss, remain to be explored [22]. A previous animal study showed that feeding a Trp-enriched diet reduced obesity via IPA [34]. In the present study, IPA levels are similar between the MTN and pretzel groups and neither fecal nor blood IPA are altered during 24 weeks intervention. IS resulting from the sulfonation of bacterially derived indole in the liver is a cardiovascular risk factor in chronic kidney disease [35]. Levels of blood IS are reduced at the end of weight loss compared to baseline in the pretzel group, but not in the MTN group, consistent with established knowledge that weight loss improves cardiovascular health [36].

Previous meta-analyses show inconsistent evidence on how caloric-restriction-based weight loss changes the gut microbiota [37,38]. In the present study, we observed that weight loss induced by moderate caloric restriction does not induce significant microbial diversity changes in neither the MTNs nor pretzel groups, as indicated by alpha and beta diversity analyses. We did, however, observe that the abundance of several bacteria previously associated with body weight and/or cardiovascular risk is altered. In the MTNs group, relative abundance of *Dubosiella* and *Muribaculum* is reduced in both weight loss (week 12) and weight maintenance (week 24) with MTNs consumption compared to baseline. In the pretzel group, relative abundance of *Tuzzerella* and *Sellimonas* is reduced, but *Methanobrevibacter* is increased in both weight loss (week 12) and weight maintenance (week 24) with pretzel consumption compared to baseline. It is worth noting that the BMI remains reduced at the end of weight maintenance (week 24) in both the pretzel and MTNs groups compared to baseline [22]. A previous study shows that *Dubosiella*, *Tuzzerella*, and *Sellimonas* have significant positive correlations with CVD pathogenesis and markers [39,40,41]. The reduction in *Dubosiella*, *Tuzzerella*, and *Sellimonas* during 24 weeks intervention is likely associated with weight loss and reduced CVD risk. Whether the effects of MTNs on *Dubosiella* contribute to the improvement in CVD risk factors (blood pressure and heart rate) needs further investigation. A previous study showed that nut consumption changed the host microbial bile acid metabolism and *Muribaculum* was previously shown to be regulated by bile acids [8,42]. Whether *Muribaculum* reduction is a consequence of MTNs consumption on bile acid metabolism is unknown. *Methanobrevibacter* is a methanogenic microbe, a risk factor for obesity by affecting caloric harvest from dietary carbohydrates [43]. Pretzels are made mostly of refined carbohydrates that offer barely any nutritional benefits and an overdose of salt. It is possible that the increase in *Methanobrevibacter* abundance is the gut microbiome’s response to caloric restriction by enhancing its caloric harvest from a dietary carb source. In addition, we only identified changes in relative abundance between baseline and week 12 of genus *Paludicola* that significantly differ between the MTNs and pretzel groups. The role of *Paludicola* in human metabolic health remains unknown. 

Growing evidence indicates that Trp and gut microbiota interactions are involved in many aspects of human physiological processes [9]. Many microbes involved in Trp metabolism have been identified and studied [9]. Here, we explored the association between microbes and Trp metabolism in overweight/obese individuals. As we expected, the most significant associations, both negative and positive, are identified between microbes and fecal Trp and indole metabolites, since the GI tract is where interactions between dietary Trp and microbes occur. In agreement with a previous study showing that *Ruminococcus gnavus* is a tryptamine producer, and we observe significantly positive association between *Ruminococcus gnavus* and fecal tryptamine [31]. *Defluviitaleaceae UCG 011* is enriched in colorectal tumor mucosa microbiome [44]. We find a negative association between *Defluviitaleaceae UCG 011* and fecal IPA, which is in agreement with the reported protective effect of IPA on gut dysbiosis [45].

The significant amount of cross-talk between host and gut microbiome with regard to Trp metabolism has been previously reported. Here, we report the potential altering of the Trp metabolism by MTN intake. Surprisingly, we do not detect bacterial composition changes between intervention groups during weight loss. Whether the effects of MTN consumption and caloric restriction on Trp microbial metabolism results from altered microbial function or complex microbiome–host interaction remains to be explored.

## Data Availability

Data is contained within the article and Supplementary Material.

## Supplementary Materials

The following supporting information can be downloaded at: https://www.mdpi.com/article/10.3390/nu15030569/s1; Table S1: Participants’ characteristics at baseline; Table S2: Multivariable association between bacterial genera and Trp metabolites.
