# Supplementary material for: Mixed Nuts as Healthy Snacks: Effect on Tryptophan Metabolism and Cardiovascular Risk Factors
Source: Nutrients. 2023 Jan 21;15(3):569. doi: 10.3390/nu15030569 (PMC9921623; doi:10.3390/nu15030569)
Supplement: Supplementary file 1 [file nutrients-15-00569-s001.zip › nutrients-2149904-supplementary.pdf]

Supplementary Table S1. Participants' characteristics at baseline.

	MTNs (n=56)	Pretzel (n=39)	P value
Age	48.3 (14.1)	46.8 (10.7)	0.76
Female (%)	69.6%	84%	0.23
Body weight (lb)	192.2 (28.3)	183.8 (30.1)	0.16
BMI	31.1 (2.6)	30.7 (2.4)	0.43

Supplementary Table S2. Multivariable association between bacterial genera and Trp metabolites.

feature	value	coef	pval	qval	-log(qval)*sign(coef)
Slackia	B_IPA	0.409	0.000	0.045	0.551
Olsenella	B_IPA	0.349	0.000	0.060	0.425
Escherichia Shigella	B_IS	0.756	0.001	0.098	0.761
Coprococcus	B_IS	0.353	0.001	0.100	0.352
Prevotella_7	B_IS	0.206	0.001	0.150	0.170
Angelakisella	B_IS	0.202	0.003	0.209	0.137
GCA 900066575	B_IS	-0.291	0.004	0.231	-0.185
Fournierella	B_KYN	-0.229	0.001	0.149	-0.190
Frisingicoccus	B_KYN	-0.198	0.003	0.232	-0.126
Candidatus Soleaferrea	B_KYN/Trp ratio	0.187	0.001	0.101	0.186
Eisenbergiella	B_KYN/Trp ratio	0.355	0.002	0.179	0.265
Frisingicoccus	B_KYN/Trp ratio	-0.217	0.002	0.179	-0.162
Dubosiella	B_Serotonin	0.292	0.000	0.039	0.410
Acidaminococcus	B_Serotonin	0.240	0.000	0.055	0.303
Mogibacterium	F_IAA	0.118	0.002	0.205	0.081
Blautia	F_Indole	0.141	0.001	0.077	0.157
Prevotella	F_Indole	-0.335	0.002	0.173	-0.255
Family XIII AD3011 group	F_IPA	-0.556	0.000	0.001	-1.640
Defluviitaleaceae UCG 011	F_IPA	-0.415	0.000	0.006	-0.921
Haemophilus	F_IPA	0.508	0.000	0.027	0.801
UCG 002	F_IPA	-0.418	0.000	0.060	-0.512
UBA1819	F_IPA	-0.371	0.002	0.148	-0.308
Anaerotruncus	F_IPA	-0.225	0.003	0.187	-0.164
Butyricicoccus	F_IPA	0.389	0.004	0.216	0.259
Dorea	F_Serotonin	-0.185	0.001	0.122	-0.169
Blautia	F_Serotonin	-0.113	0.002	0.194	-0.080
Solobacterium	F_Serotonin	0.134	0.003	0.216	0.089
Lachnospiraceae ND3007 group	F_Trp	-0.669	0.000	0.003	-1.703
Candidatus Stoquefichus	F_Trp	0.230	0.000	0.005	0.536
Eggerthella	F_Trp	0.364	0.000	0.009	0.746
Flavonifractor	F_Trp	0.452	0.000	0.009	0.927
UCG 005	F_Trp	-0.602	0.000	0.014	-1.125
Eubacterium xylanophilum group	F_Trp	-0.501	0.000	0.015	-0.913
Ruminococcus gnavus group	F_Trp	0.488	0.000	0.018	0.856
Anaerostipes	F_Trp	0.318	0.000	0.018	0.556
Lachnospira	F_Trp	-0.592	0.000	0.018	-1.035
Parabacteroides	F_Trp	-0.534	0.000	0.018	-0.935
Blautia	F_Trp	0.148	0.000	0.022	0.244
Erysipelotrichaceae UCG 003	F_Trp	-0.440	0.001	0.058	-0.545
Odoribacter	F_Trp	-0.436	0.001	0.079	-0.480
Erysipelatoclostridium	F_Trp	0.425	0.001	0.089	0.446
Holdemania	F_Trp	0.244	0.002	0.128	0.218
Lachnospiraceae UCG 004	F_Trp	-0.333	0.003	0.128	-0.298
Sutterella	F_Trp	-0.415	0.002	0.128	-0.371
Faecalitalea	F_Trp	0.299	0.006	0.221	0.196
Tuzzerella	F_Trp	0.181	0.006	0.221	0.119
Barnesiella	F_Trp	-0.336	0.007	0.240	-0.208
Ruminococcus gnavus group	F_Tryptamine	0.510	0.000	0.044	0.690
Faecalibacterium	F_Tryptamine	-0.309	0.001	0.117	-0.287
Christensenella	F_Tryptamine	0.123	0.001	0.117	0.115
Megasphaera	F_Tryptamine	-0.242	0.003	0.222	-0.158
Rikenellaceae RC9 gut group	F_Tryptamine	0.236	0.004	0.241	0.145
